# Investigating the effect of immunomagnetic separation on the immunophenotype and viability of plasma cells in plasma cell disorders

**DOI:** 10.3389/pore.2024.1611882

**Published:** 2024-10-18

**Authors:** Ágnes Czeti, Soma Sashalmi, Ferenc Takács, Gábor Szalóki, Csilla Kriston, Gergely Varga, Péter Farkas, Aryan Hamed, Ágnes Márk, Gábor Barna

**Affiliations:** ^1^ Department of Pathology and Experimental Cancer Research, Semmelweis University, Faculty of Medicine, Budapest, Hungary; ^2^ Department of Internal Medicine and Haematology, Semmelweis University, Faculty of Medicine, Budapest, Hungary; ^3^ Department of Haematology, Aladar Petz County Teaching Hospital, Győr, Hungary

**Keywords:** multiple myeloma, flow cytometry, immunomagnetic separation, immunophenotype, positive selection

## Abstract

Plasma cell enrichment plays a pivotal role in the accurate prognosis and molecular characterization of multiple myeloma. The separation is commonly carried out by positive cell selection using CD138 monoclonal antibody conjugated to magnetic beads. Optimally, during the separation procedure, the cells should neither be damaged, nor should their phenotype be significantly altered, as these changes would falsify the results if the isolated cells were subsequently used. For this reason, we investigated the expression patterns of different surface markers by flow cytometry before and after magnetic isolation using bone marrow or peripheral blood samples from 12 patients with plasma cell disorders. The selected markers are not only used as backbone markers in routine diagnostics (CD19, CD38, CD45, CD117, and CD138), but they also play an important role in cell adhesion and connection with microenvironment (CD44, CD49d, CD56, and CD81) or possibly drug resistance (CD69, CD86, and CD184), making them promising targets for myeloma research. Moreover, we examined the effects of separation on cell viability in 8 cases. The intensities of 8 out of the 12 investigated markers were slightly influenced, while CD138, CD38, CD56, and CD184 were changed significantly, however the immunophenotype of the cells was not changed. Positive markers remained positive and negative ones remained negative after the separation procedure. In addition, the number of apoptotic plasma cells was significantly reduced during separation, facilitating further examination of the cells. Our results showed that magnetic isolation can be considered as a reliable option but the immunophenotype of plasma cells should be validated after the separation if the intensities of the markers are important for further experiments.

## Introduction

Multiple myeloma (MM) is a currently incurable malignant disease of plasma cells. It is the second most common hematologic malignancy, accounting for more than 15% of all hematologic tumors in the United States [1]. In this disease, myeloma cells can be present in various proportions in both bone marrow (BM) and peripheral blood (PB) [2].

Low percentage of tumor cells and the presence of other, non-malignant cells in the samples may make the diagnostic procedures (e.g., fluorescence *in situ* hybridization) or the research of the molecular mechanisms of the disease (e.g., gene expression profiling) more challenging [3–6]. Therefore, the enrichment of the plasma cells is crucial to detect chromosomal aberrations or studying myeloma cells at the molecular or protein level.

One of the most commonly used methods is immunomagnetic isolation, which can be used to produce a highly pure, homogeneous cell population quickly and in a relatively cost-efficient way. This technique is based on antibodies conjugated to magnetic beads that bind a marker on the surface of the target cells we aim to enrich. Then, by placing the sample in a magnetic field, the magnetically labeled cells are separated from the other cells (positive selection). Purification of myeloma cells is often performed by this method using anti-CD138 monoclonal antibodies conjugated to magnetic beads, since plasma cells, in contrast to other cells in the bone marrow, express high levels of the proteoglycan CD138 (syndecan-1) [7–9].

It is crucial to maintain the phenotype of the cells unaltered throughout the separation procedure, as any modification could significantly skew the results. This is particularly important in experiments assessing the presence of surface markers.

Despite the potentially serious consequences, we have only found limited studies from a single research group in the literature [10, 11] that investigated the effects of magnetic separation on the immunophenotype of myeloma cells. In their most recent research, Bansal et al. reported a significant decrease in several surface markers (e.g., CD138, CD11a, CD49e, and CD69) as a result of separation.

Therefore, in this paper, we aimed to study and validate whether CD138-based immunomagnetic separation affects the phenotype or viability of myeloma cells. We examined the protein expression patterns of various surface markers by flow cytometry before and after isolation. The studied markers are of great importance in clinical research as they are either used as backbone markers in routine diagnostics (CD19, CD38, CD45, CD117, and CD138) [12], involved in cell adhesion and connection with microenvironment (CD44, CD49d, CD56, and CD81) [13–15], or possibly in drug resistance (CD69, CD86, and CD184) [16–19].

For this reason, it is crucial to investigate whether and how the expression of these markers changes during immunomagnetic separation.

## Materials and methods

### Patient samples

We analyzed 12 samples (11 BM from myeloma and 1 PB from plasma cell leukemia patients) by flow cytometry. The samples were collected in EDTA (ethylenediamine tetraacetic acid) tubes. A total of seven women [median age: 64 years (50–79)] and five men (median age: 69 years [53–86]) were enrolled in the study. The clinical parameters of the samples are summarized in Supplementary Table S1. The diagnoses were made according to the recommendations of the World Health Organization [20] and the International Myeloma Working Group (IMWG) [21]. Following the recommendations of J. Cumova et al. [7], all of our samples contained at least 5% plasma cells (PC) among all BM or PB cells. The mean percentage of malignant plasma cells was 26.6% [9.3%–56.3%] relative to all cells in the samples. All samples were processed within 1 day of collection. The study was conducted in accordance with the Declaration of Helsinki and approved by the local ethics committee (ethical approval number: SE RKEB: 214/2020).

### Immunomagnetic separation of CD138+ cells

Plasma cells were enriched from samples using the EasySep™ Human Whole Blood and Bone Marrow CD138 Positive Selection Kit II (StemCell Technologies, Vancouver, BC, Canada). First, samples (at least 1 mL of bone marrow or peripheral blood), were filtered through a 40 µm filter, then red blood cells were lysed without fixation (BD Pharm Lyse™, Becton Dickinson (BD) Bioscience, New Jersey, United States), as it is crucial to maintain the integrity of the cells and the right conformation of surface proteins. After removing the lysing solution and washing with phosphate-buffered saline (PBS) (pH = 7.4, 300 g, 10 min with the brake off), EasySep Buffer was added to the pellets. Then, following the manufacturer’s protocol [22], cells were mixed with the positive selection cocktail containing anti-CD138 antibodies. After incubation, magnetic particles were added to the samples. Finally, the separation tubes were placed in the EasySep™ magnet for 10 min. After the purification steps, isolated plasma cells were obtained. The purity of the homogeneous plasma cell population was confirmed by flow cytometry where plasma cells were identified by CD45/CD38 and CD138 markers (for details, see Cell surface staining protocol section). The gating strategy used to verify the purity of the separated samples is described in detail in Supplementary Figure S1.

### Flow cytometry analysis before and after magnetic cell separation

#### Cell surface staining protocol

The myeloma cell ratio (%) was determined by flow cytometry in a preceding measurement, wherein 10^6^ cells were stained by the Panel 1 (Table 1) in 50 µL total volume and plasma cell ratio were assessed. The plasma cell concentrations of samples were set to 0.25 × 10^6^/samples for immunophenotype determination before and after separation. Samples were stained with fluorochrome-conjugated monoclonal antibodies against the following surface antigens: CD19, CD38, CD44, CD45, CD49d, CD56, CD69, CD81, CD86, CD117, CD138, and CD184 (see Table 1 for details). The samples were incubated with the antibodies for 13 min at 4°C. After incubation, we lysed the red blood cells with FACSLysing solution (BD Bioscience), containing fixation agent (paraformaldehyde). This lysis step was applied both on the separated and unseparated plasma cells to keep the experimental conditions identical. The lysing solution was removed by centrifugation (400 g, 10 min, room temperature (RT)). Then, we washed the cells with PBS using the same settings (400 g, 10 min, RT). Afterwards, the fluorescent nucleic acid dye SYTO-40 (Invitrogen, Waltham, United States) was added to the collected cells. 10,000–150,000 events were measured per sample using a 10-color BD FACSLyric™ flow cytometer (BD Bioscience). The cytometer was regularly calibrated with CS&T Beads (BD Bioscience). Myeloma cells were identified by their high CD38/CD138 expression and low CD45 levels.

**TABLE 1:** Overview table of antibodies used in our experiments.

*Laser*	*Violet* 405nm, 40 mW			*Blue* 488nm, 40 mW				*Red* 640nm, 40 mW
*Fluorescent channels*	FL1	FL2	FL3	FL4	FL5	FL6	FL7	FL8
*Filters*	448/45	528/45	606/36	527/32	586/42	700/54	783/56	660/10
*Panel 1*	Syto40	CD138 BV510 (MI15, Sony)	CD45 BV605 (HI30, Sony)	CD81 FITC (JS-81, BD)	CD56 PE (C5.9, Dako)	CD19 PERCP-Cy5.5 (HIB19, Sony)	CD117 PC7 (104D2D1, BC)	CD38 APC (LS198-4–3, BC)
*Panel 2*	Syto40	CD138 BV510 (MI15, Sony)	CD45 BV605 (HI30, Sony)	CD69 FITC (FN50, BD)	CD38 PE (AT13/5, Dako)	CD44 PERCP-Cy5.5 (IM7, Sony)		CD86 APC (2331-(FUN-1), BD)
*Panel 3*	Syto40	CD138 BV510 (MI15, Sony)	CD45 BV605 (HI30, Sony)	CD38 FITC (AT13/5, Dako)	CD184 PE (12G5, BD)			CD49d APC (9F10, Sony)

Summary of the antibodies used in our experiment to investigate cell phenotypes before and after immunomagnetic separation, using a 10-color BD FACSLyric™ flow cytometer. (BC, beckman coulter; BD, becton dickinson; APC, allophycocyanin; BV, Brilliant Violet™; FITC, fluorescein isothiocyanate; PC7, Phycocyanin-7; PE, phycoerythrin; PERCP-Cy5.5, Peridinin-chlorophyll-Cyanine5.5).

In this experiment, the immunophenotype of 12 samples was examined. Due to the large number of antibodies involved, we used three panels during the procedure (Panel 1, Panel 2, Panel 3). Each panel contained conventional myeloma backbone markers (Table 1). Panel 2 and Panel 3 were applied on all 12 samples, while Panel 1 was used on 8 of them.

All antibodies were pre-titrated and isotype controls were used for most antibodies to exclude non-specific binding. The relative median fluorescence intensity (rMFI) values were calculated by subtracting the MFI of the isotype controls from the MFI values associated with the markers. The gating strategy is described in detail in Supplementary Figure S2.

### Measurement of viability with propidium-iodide

In 8 cases we also investigated the effect of separation on the viability of plasma cells. For this purpose, we compared the proportion of propidium-iodide-positive cells (PI+) among all plasma cells before and after separation. In this experiment, CD38-FITC (DAKO, Agilent, Santa Clara, United States) antibody was used to identify the plasma cells. After cell surface labeling (13 min, 4°C), the samples were gently lysed with an ammonium chloride-based lysing reagent (BD Pharm Lyse™). The lysing solution was removed by centrifugation (400 g, 10 min, RT) and the samples were washed with PBS (400 g, 10 min, RT). Cells were then stained with propidium-iodide (2 µg/10^5^ cells) (PI, Sigma). Samples were measured on an 8-color Navios flow cytometer (Beckman Coulter (BC), Brea, California, United States) after the final washing step. The cytometer is regularly calibrated with Flow-Check Pro and Flow-Set Pro QC beads (BC). The gating strategy is described in detail in Supplementary Figure S3.

### Data analysis and statistics

Flow cytometric data were analyzed with the Kaluza 2.1.1 software (Beckman Coulter, Brea, CA, United States). The values used in our statistics are either MFI, or the percentage of PI-positive cells compared to all plasma cells in the sample. We used SigmaPlot 12.5 (Systat Software Inc., San Jose, California) for plotting and statistical analysis. Normality (Shapiro-Wilk) and equal variance tests were performed in each case, followed by paired t-test and the Wilcoxon signed-rank test to determine statistical significance with p < 0.05.

## Results

### Purity of plasma cell isolation

In this study, we investigated the effect of CD138 antibody-based immunomagnetic separation on plasma cells in 12 samples from patients with plasma cell disorder.

**FIGURE 1:**
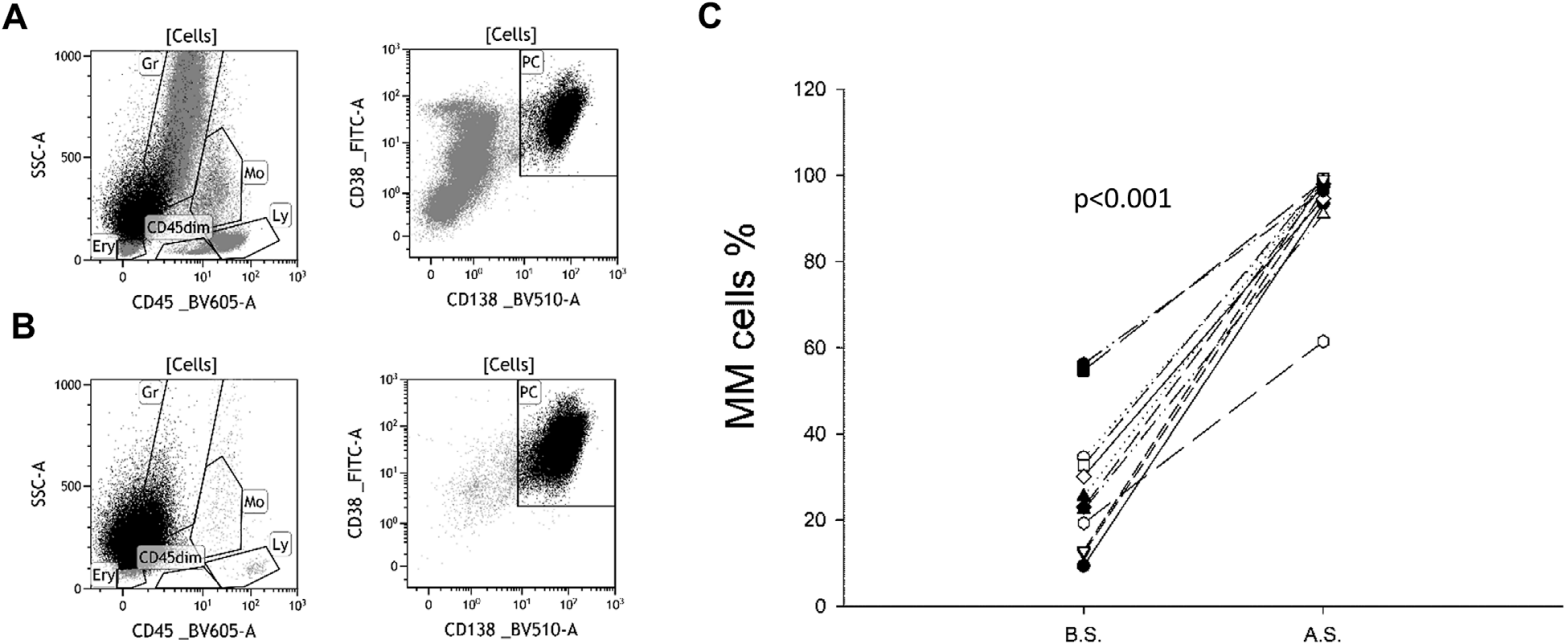
Plasma cell ratio before and after separation. CD138-CD38 dot plots of a representative sample before **(A)** and after **(B)** separation. Originally, plasma cells (PC) accounted for 23.4% of all cells. As a result of magnetic separation, the vast majority of non-plasma cells (grey) were removed, increasing the percentage of PC to 95.2%. A similar trend was observed in all 12 samples **(C)**. B.S. means before separation, A.S. means after separation.

The purity of the plasma cell population after isolation ranged from 61.46% to 99.22% with a mean of 93.74% and a standard deviation (SD) of 10.48 (Figure 1).

### Examination of changes in cell surface markers

The immunophenotypes of 12 samples were studied. In eight of these, we examined 12 surface markers commonly used in myeloma research, while in four cases we studied only a subset of them (CD38, CD44, CD45, CD49d, CD69, CD86, CD138, and CD184).

In our experiments, we compared the median fluorescence intensities (MFI) of the malignant plasma cell population before and after magnetic separation in the samples in which the myeloma cells formed a homogeneous population. In a few samples for certain surface markers, multiple subpopulations of myeloma cells with different expression levels were observed, see Table 2. The results of these samples were evaluated separetly from those with homogeneous populations.

**TABLE 2:** Overview of cases with distinct subpopulations. Changes in the MFI values of the subpopulations and in their relative ratio before (B.S.) and after the separation (A.S.) are shown. Relative MFI values are marked with *italic*. Results with significant changes are marked with **bold**.

*Cell surface marker*	*Patient Sample*	*Dimmer subpopulation B.S.*		*Dimmer subpopulation A.S.*		*Brighter subpopulation B.S.*		*Brighter subpopulation A.S.*	
		%	MFI	%	MFI	%	MFI	%	MFI
***CD38***	***Sample 4***	43.87	75.88	50.59	82.17	56.13	199.26	49.41	237.22
***CD44***	***Sample 4***	**10.22**	*−0.54*	**12.97**	*−6.06*	**89.78**	*111.01*	**87.03**	*128.51*
	***Sample 10***	**58.03**	*0.56*	**63.28**	*−3.58*	**41.97**	*59.93*	**36.72**	*64.54*
	***Sample 11***	**35.05**	*2.67*	**40.31**	*−2.91*	**64.95**	*35.12*	**59.69**	*41.57*
	***Sample 12***	**21.67**	*0.12*	**26.02**	*0.67*	**78.33**	*115.74*	**73.98**	*149.46*
***CD45***	***Sample 4***	40.64	1.21	44.4	0.99	59.36	7.53	55.6	7.01
	***Sample 10***	26.13	1.64	22.45	2.25	73.87	10.98	77.55	17.09
	***Sample 11***	89.1	1.23	83.87	1.14	10.9	9.89	16.13	1.34
***CD69***	***Sample 8***	28.51	*0.18*	25.75	*0.23*	71.49	*3.35*	74.25	*3.09*
***CD86***	***Sample 5***	14.15	*0.17*	12.93	*0.08*	85.85	*3.53*	87.07	*2.88*
***CD138***	***Sample 2***	0	0	41.34	7.3	100	19.51	58.66	45.59
	***Sample 8***	45.28	24.7	23.68	13.71	54.72	89.95	76.32	61.42
***CD81***	***Sample 10***	96.94	1.56	95.32	2.07	3.06	12.68	3.01	12.64
	***Sample 12***	8.98	1.44	5.22	1.09	91.02	7.98	94.78	11.94
***CD184***	***Sample 2***	79.91	*0.58*	69.82	*1.79*	20.09	*4.87*	30.18	*10.05*
***CD56***	***Sample 11***	73.96	3.3	71.42	2.53	26.04	31.66	28.58	44.85
***CD19***	***Sample 11***	74.53	1.6	74.02	1,91	25.47	21.38	25.98	19.61

**FIGURE 2:**
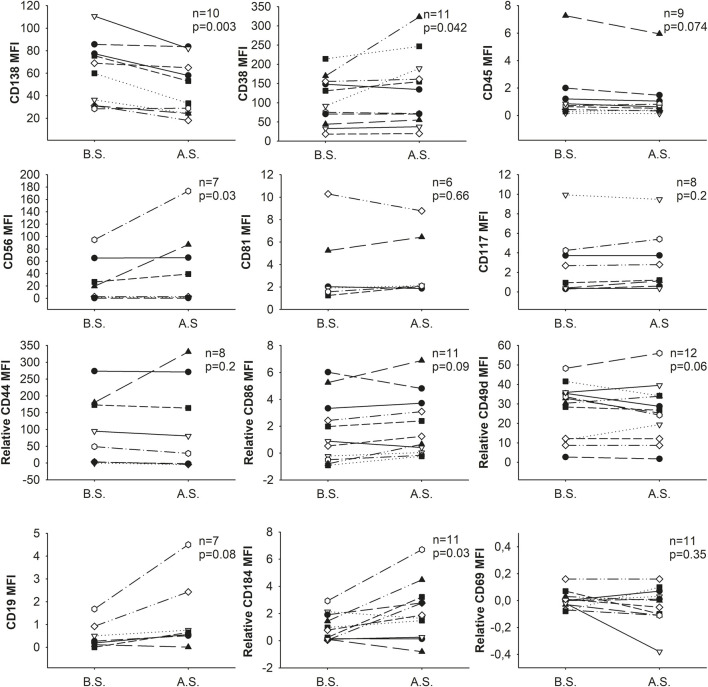
Changes in the expression (MFI: median fluorescence intensity) of cell surface markers as a result of separation. B.S. means before separation, A.S. stands for after separation.

The intensity changes of tested markers varied among different donors. In samples with homogeneously stained populations, the intensity of most tested markers (8/12) did not change significantly, and any changes observed were not consistently in one direction. Nevertheless, the intensity of CD38 (8/11 samples), CD56 (4/7 samples), and CD184 (6/11 samples) were increased, while the intensity of CD138 (9/10 samples) was decreased significantly (Figure 2).

In the case of CD38, CD56 and CD138 the change of intensity was remarkable (mean of the MFI±SD before vs. after separation, CD38: 104.7 ± 63.2 vs. 133.2 ± 94.5, p = 0.042; CD56: 30.1 ± 36.5 vs. 52.9 ± 63.2, p = 0.03; CD138: 60.5 ± 28.0 vs. 47.0 ± 24.6, p = 0.003, respectively) while in the case of CD184 the change was minor (mean of the relative MFI±SD of CD184 before vs. after separation: 0.8 ± 1.3 vs. 2.0 ± 2.3; p = 0.029).

Nevertheless, these changes did not affect the abnormal plasma cells’ phenotype, i.e., positive/negative cells remained positive/negative, respectively, when compared to isotype controls or unstained cells for CD38, CD56, CD138, and CD184 markers (Supplementary Figure S2).

**FIGURE 3:**
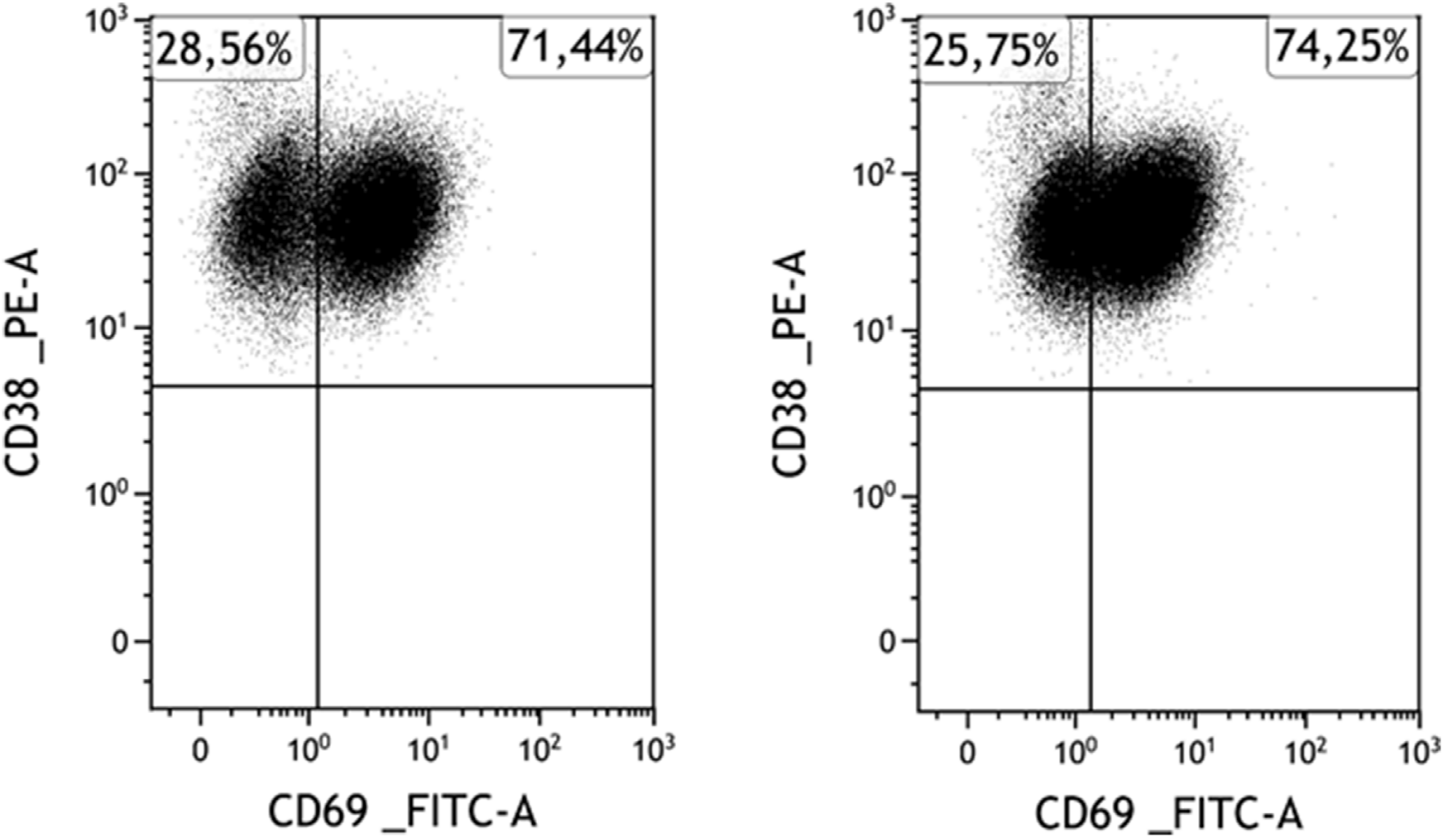
A representative case for marker expression stability: Expression of CD69 of myeloma cells before (left) and after (right) separation in a CD69-positive case. Among myeloma cells, the CD69-positive subpopulation is clearly distinguishable and intact both before and after the separation.

To examine the effects of isolation on samples with distinct subpopulations, we measured the change in MFI values of their dimmer and brighter subpopulations separately. In addition, we also examined whether the relative ratio of these subsets has changed. Samples involved and their corresponding results are shown in Table 2. Distinct populations within samples were rare, but when present, their findings mirrored those from samples with single populations. In these rare cases involving distinct populations, neither the MFI values of the subpopulations nor their relative ratios changed significantly (see Figure 3), except in the case of CD44 (mean ratio of subpopulations relative to all myeloma cells before vs. after separation, dimmer: 31.2% ± 20.5% vs. 35.6% ± 21.5, p = 0.005, brighter: 68.8% ± 20.5% vs. 64.4% ± 21.5, p = 0.0049).

In an effort to identify potential relationships between changes in intensity of tested markers and factors such as myeloma cell ratio, age or chromosomal alterations, we conducted comparisons across the examined samples. However, our analysis did not reveal any significant correlations (see Supplementary Table S1).

### Viability of plasma cells before and after separation

**FIGURE 4:**
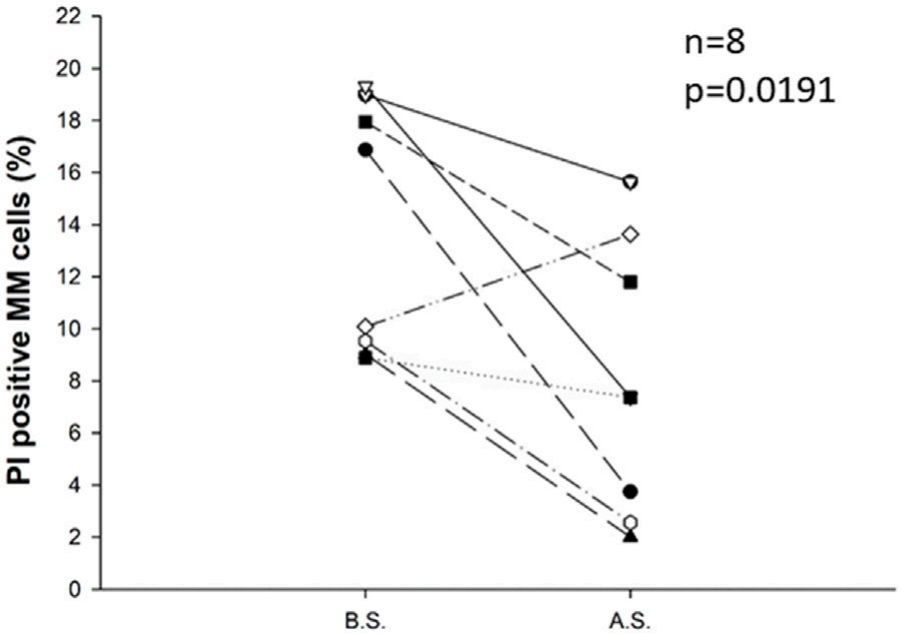
Change in the percentage of propidium-iodide-positive cells compared with all plasma cells before (B.S.) and after the separation procedure (A.S.) for eight samples. Immunomagnetic cell separation significantly decreased the percentage of PI-positive apoptotic cells in the plasma cell population (p = 0.0191). Statistical significance was set at p < 0.05.

We examined the percentage of propidium-iodide-positive cells compared with all plasma cells before and after separation in 8 cases. The results show that the ratio of dead cells in the plasma cell population was significantly reduced (from an average of 13.8% to 8.0%) during the separation procedure (p = 0.0191). Consequently, the percentage of viable plasma cells in the plasma cell population was significantly increased, see Figure 4.

## Discussion

Enrichment and purification of the malignant population is a critical step to perform molecular experiments and for the clinical research of multiple myeloma. One of the most suitable methods for this purpose is the positive immunomagnetic cell separation based on the CD138 positivity of myeloma cells. However, procedural conditions and environmental changes can lead to cell damage, molecule shedding, or the expression of previously unexpressed proteins [10, 23], potentially altering the phenotype of the cells and significantly affecting the outcomes of *ex vivo* or *in vitro* experiments.

In our study, we observed slight changes in the intensity of most tested markers following magnetic separation, but significant differences in the expression patterns of the majority of proteins (CD44, CD45, CD49d, CD69, CD81, CD86, CD117, and CD19) were not detected. Notably, only the intensities of CD38, CD56, and CD184 were significantly increased, whereas the intensity of CD138 significantly decreased during the separation process. Contrary to the findings of Bansal et al. [10], who observed significant reductions in the expression levels of various cell surface markers (including CD11a, CD11b, CD49e, CD69, CD71, and CD138) following magnetic separation with the same kit, our results do not align, especially regarding CD69 and CD138. In our study, CD69 expression remained consistent, showing no significant change (Figures 2, 3). While CD138 (syndecan-1) was basically absent from the cell surface after Bansal’s isolation procedure, we observed only a slight decrease in the fluorescence intensity of CD138.

This slight decrease could be attributed to technical factors, such as the blockage caused by residual attached antibodies following the separation process. The reason can also be biological, including shedding, potentially triggered by various conditions like cellular activation or stress (e.g., hyperosmolarity) experienced during the isolation process [24].

In addition to shedding, the observed reduction in CD138 expression might be explained by an increase of apoptotic cells, as it is established that dead cells exhibit diminished levels of CD138 [25]. To test whether an elevated proportion of apoptotic cells during the separation process contributed to the reduced CD138 expression, we assessed the ratio of apoptotic and necrotic (propidium-iodide, or PI-positive) cells before and after isolation. As expected and previously demonstrated in the literature [26], the ratio of dead cells decreased following the separation process, likely because apoptotic cells, which have lower CD138 levels, are washed away. Thus, we can conclusively state that apoptotic or necrotic cells were not responsible for the decrease in CD138 levels observed.

In our samples, we observed increased staining of CD38, CD56, and CD184. However, only the CD38 levels were systematically elevated in most of the samples.

The observed phenomenon might be due to the samples being predominantly composed of myeloma cells, thereby attracting more antibodies, even though the antibodies for CD38 detection were carefully titrated before use. Since CD38 expression is easily inducible on immun cells [27], an increase in their levels may also be caused by changes in the cytokine environment during isolation.

Providing a clear explanation for the changes in intensity of the other two molecules proves challenging. The intensity of CD56 significantly increased in only half of the samples, demonstrating inconsistency. In the case of CD184, while the intensity changing were statistically significant, the absolute values changed minimally in the samples.

In a few cases, multiple subpopulations of the myeloma cells occurred during our experiments for some of the surface markers. For these samples, we measured the change in MFI values of these dimmer and brighter subpopulations separately and found that they did not change notably, similar to samples with a single population. To further study the potential changes in heterogenous samples, we also examined whether their relative ratio compared to all myeloma cells changed (see Table 2). However, except for the case of CD44, no significant change occurred.

Additionally, we have explored the possibility that changes in marker intensity post-separation could be linked to factors such as cytogenetic abnormalities, age, or myeloma cell ratio. However, our investigations revealed no association between these factors and the alterations in marker expression observed after the separation process.

The limitations of our study may arise from using only 12 samples, which could narrow the scope of our conclusions. To address this concern, we standardized the staining procedures across all samples, ensuring each was analyzed with an identical number of plasma cells.

## Conclusion

In this study we examined the expression pattern of several cell surface markers that are important for the diagnosis and progression of multiple myeloma before and after magnetic isolation. We found in our experiments that in the vast majority of cases, immunomagnetic separation may slightly change the intensity of tested markers of plasma cells but does not change the immunophenotype of the cells. Positive markers remained positive and negative ones remained negative after the separation procedure. Still, it is recommended to validate the immunophenotype of the isolated cells after magnetic separation in experiments where the marker intensity of the cells or the amount of cell surface proteins are particularly important.

## Data Availability

The raw data supporting the conclusions of this article will be made available by the authors, without undue reservation.
