# Supplementary material for: Investigating the effect of immunomagnetic separation on the immunophenotype and viability of plasma cells in plasma cell disorders
Source: Pathol Oncol Res. 2024 Oct 18;30:1611882. doi: 10.3389/pore.2024.1611882 (PMC11527611; doi:10.3389/pore.2024.1611882)
Supplement: Supplementary file 1 [file DataSheet1.docx]

Suppl. Table 1. Clinical characteristics of patients.

| Sample number | Age (years) | Tissue type | MM cell ratio (%) | Chromosomal aberrations | Disease status | Intensity changes |
| --- | --- | --- | --- | --- | --- | --- |
| Sample 1 | 65 | BM | 13 | 1q21 ampl | newly diagnosed | CD38, CD138, CD49d |
| Sample 2 | 86 | BM | 56 | n.a. | newly diagnosed | CD38, CD138, CD49d |
| Sample 3 | 70 | BM | 21 | del17p (7%) ^a^ | newly diagnosed | CD38, CD138, CD86, CD184 |
| Sample 4 | 78 | BM | 9 | 1q21 ampl (12%) | newly diagnosed | CD38, CD138, CD44, CD86, CD184 |
| Sample 5 | 55 | BM | 33 | t(14;16) | newly diagnosed | CD38, CD138, CD49d |
| Sample 6 | 53 | BM | 17 | no aberrations | newly diagnosed | CD38, CD138, CD44, CD49d CD56, CD81, CD117, CD184 |
| Sample 7 | 79 | BM | 13 | 3 IgH signals, del17p (35%), 1q21 ampl, t(14;16) | newly diagnosed | CD38, CD138, CD86, CD117 |
| Sample 8 | 59 | BM | 38 | Monosomy of chromosomes 1 and 17 | newly diagnosed | CD38, CD138, CD44, CD49d, CD86 |
| Sample 9 | 69 | BM | 24 | t(4;14) | 3^rd^ relapse* | CD38, CD138, CD56, CD81, CD44, CD49d |
| Sample 10 | 64 | BM | 34 | 3 IgH signals, t(11;14) | newly diagnosed | CD38, CD138, CD56, CD81, CD117, CD184 |
| Sample 11 | 50 | BM | 25 | 3 IgH signals, t(11;14) | newly diagnosed | CD38, CD138, CD44, CD56, CD184 |
| Sample 12 | 62 | PB | 35 | 3 IgH signals, 1q21ampl, t(11;14) | newly diagnosed | CD38, CD138, CD44, CD56, CD184 |

BM: bone marrow, PB: peripheral blood, n.a.: not available, amp: amplification, del: deletion, t(xx;xx): translocation, IgH: Immunoglobulin Heavy Chain

MM cell ratio was determined by flow cytometry in BM or PB, Chromosome aberrations determined by Fluorescent In Situ Hybridization (FISH).

Markers with intensity change exceeding 10% are listed in column 7.

^a^ The percentage of cells affected by the mutation.

*The patient did not receive any treatment in the 9 months preceding the bone marrow aspiration.


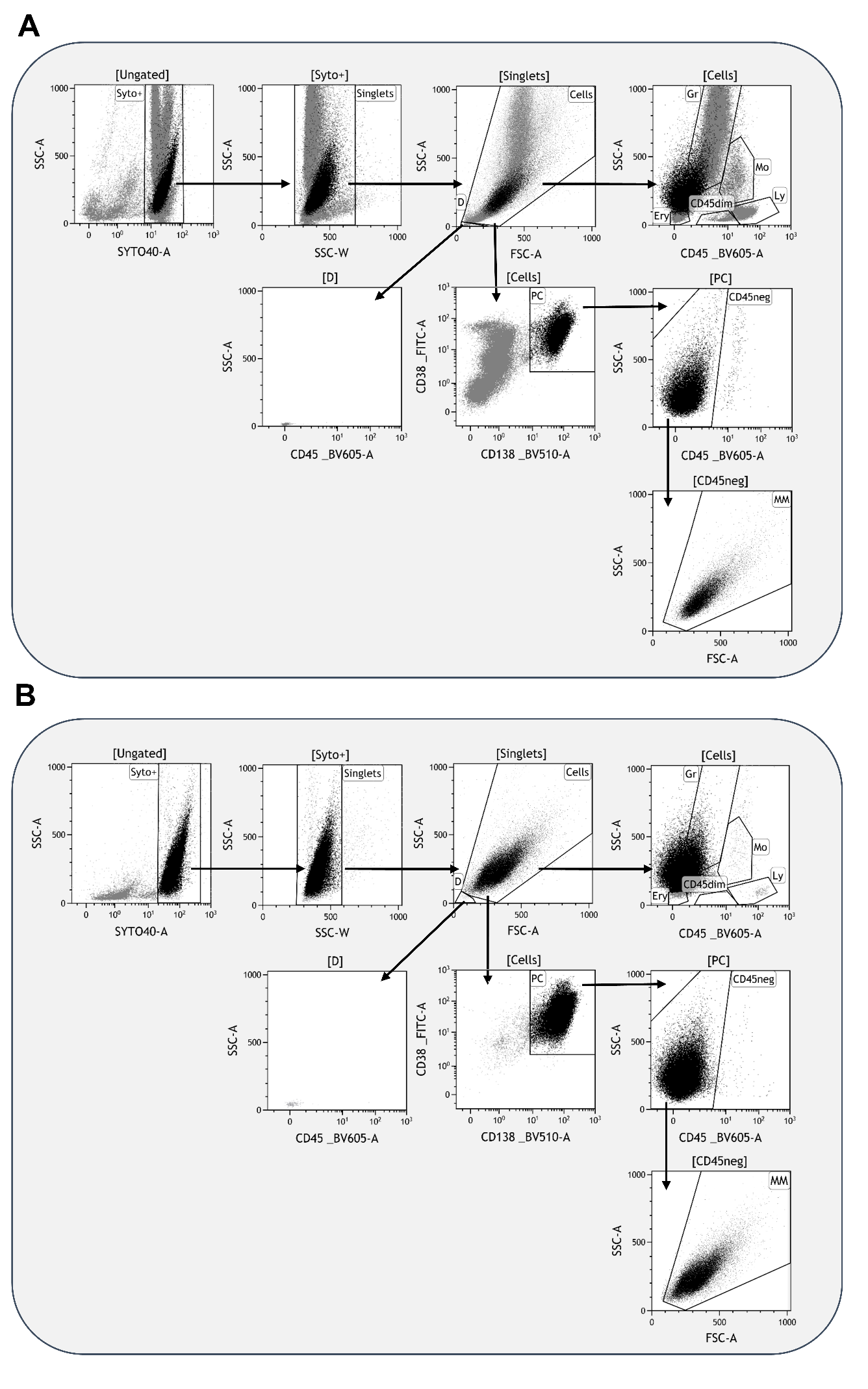
Supplementary Figure 1.

Depiction of the gating strategy used to verify the purity of the samples after separation.

A: We show the gating steps applied to the sample before its separation: After identifying the nucleated cells (Syto+), we excluded doublets (Singlets) and debris (D). SSC-CD45 dot plot of the remaining cells (Cells gate) shows plasma cells in black, while all others are shown in grey (Gr-granulocytes, Mo-monocytes, Ly-lymphocytes, CD45dim-blasts/hematogones, Ery-erythrocytes). Plasma cells (PC) were gated based on their high CD38 and CD138 expression. A backgating analysis was performed for CD45-SSC and FSC-SSC to verify the remaining debris.

B: Verifying the purity of the sample after isolation. Similar to “A”, we first identified the Syto positive, singlet cells (Singlets gate). Next, we removed the debris (D gate). The SSC-CD45 dot plot of the removed debris shows that there are no further cells in D gate. The remaining cells in Cells gate are shown in both SSC-CD45 and CD38-CD138 dot plots. Finally, plasma cells (PC) were gated based on their high CD38 and CD138 expressions. Backgating analysis was performed for FSC-SSC to verify the absence of remaining debris.


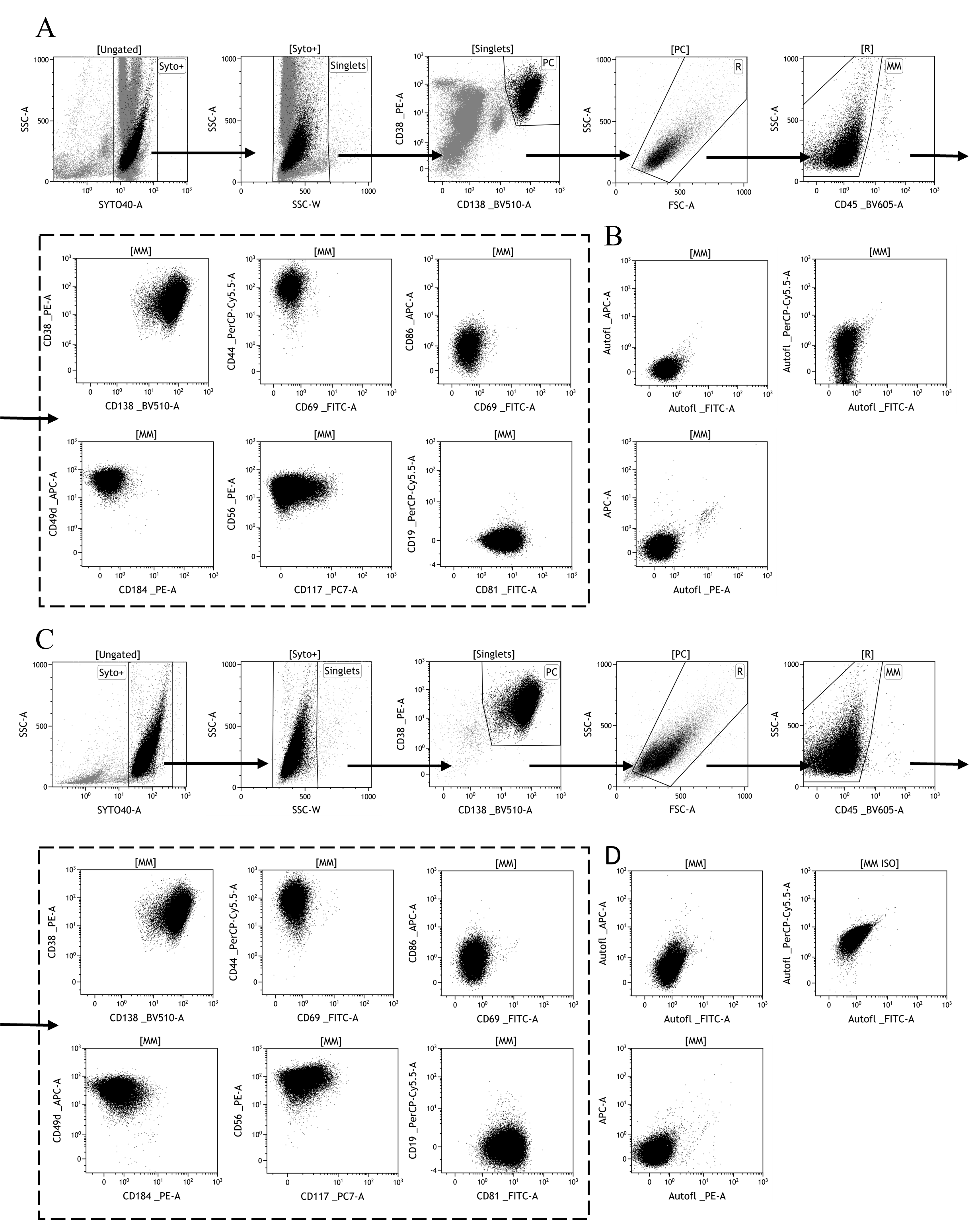


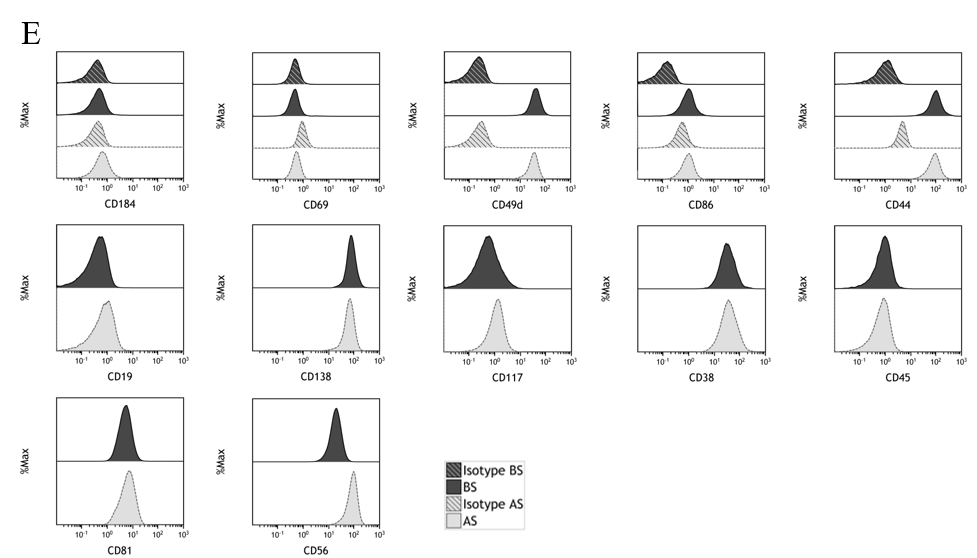


Supplementary Figure 2.

Depiction of the gating strategy used to compare the surface stainings of the samples before and after separation.

A: We present the gating steps applied to the sample before its separation: After identifying the nucleated cells (Syto+), we excluded doublets (Singlets). The plasma cells (PC)(black) were gated based on their high CD38 and CD138 expression, followed by backgating to FSC-SSC and CD45-SSC to exclude debris. On these purified myeloma cell populations (MM) the relative expressions of surface markers were determined by using isotype controls (B).

C: After separation the same gating steps were applied to identify the myeloma cells (MM). Isotype controls (D) were used to determine the relative expression of the investigated markers.

E: Representative histograms depicting the expression levels of selected CD markers in a single sample before (BS) and after separation (AS), alongside isotype controls. The histograms illustrate the changes in marker expression due to the separation process.


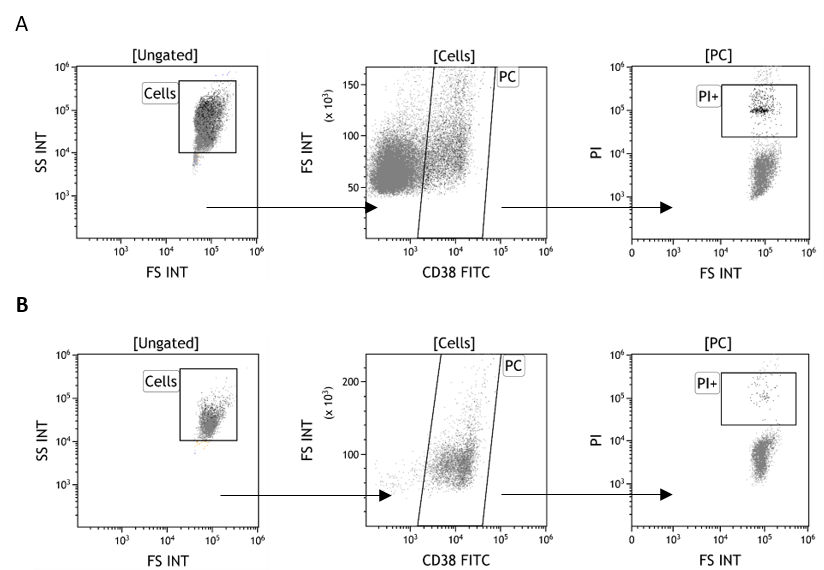


Supplementary Figure 3. Study the viability of myeloma cells with propidium-iodide staining. We measured the amount of PI-positive (PI+) cells among all plasma cells before (A) and after (B) magnetic separation. The cells (Cells gate) were first separated from debris using forward (FS INT) and side scatters (SS INT). The plasma cells (PC gate) were gated based on their high CD38 expression. Apoptotic and necrotic cells were intensively marked by propidium-iodide staining (PI+ gate). In the presented sample, separation reduced the number of PI+ plasma cells from 9.1% to 1.7%.
